# Rumen Microbiota and Metabolome Reveal the Effects of *Yucca schidigera* Extract on Growth Performance and Serum Immune Parameters in Fattening Goats

**DOI:** 10.3390/ani16193020

**Published:** 2026-09-25

**Authors:** Hongna Wang, Hui Zhao, Chenchong Pan, Jiaoyang A, Zepeng Duan, Erjun Ren, Xiaolong Wu, Siying Meng, Jie Liu, Jiaxin Chen

**Affiliations:** 1 College of Life Sciences and Food Engineering, Hebei University of Engineering, Handan 056000, China; wanghongna08@163.com (H.W.); hx178011@163.com (H.Z.); 15930866323@163.com (C.P.); 18303082221@163.com (J.A.); 17636469271@163.com (Z.D.); wxl18835202428@163.com (X.W.); 18533255106@163.com (S.M.); 2 Shijiazhuang Academy of Agriculture and Forestry Sciences, Shijiazhuang 050051, China; renerjun2012@163.com (E.R.)

**Keywords:** *Yucca schidigera* extract, fattening goats, growth performance, rumen microbiota, metabolome

## Abstract

This study aimed to investigate the effects of *Yucca schidigera* extract (YSE) on growth performance, serum immune parameters, rumen microbiota, and metabolites of fattening goats. For this purpose, thirty-two healthy male Boer goats with similar initial body weights (36.91 ± 3.05 kg) were randomly assigned to four groups. Goats in the control group (Group A) were fed a basal pelleted diet, whereas the experimental groups (Groups B, C, and D) received 200, 400, and 600 mg of YSE per kg of basal diet, respectively. The results reveal that dietary supplementation with 600 mg/kg YSE significantly elevated average daily gain (ADG) and decreased feed-to-gain ratio (F/G) (*p* < 0.05). The 400 mg/kg YSE addition significantly increased serum IgM concentration and reduced the TNF-α concentration (*p* < 0.05). Meanwhile, 400 mg/kg dietary YSE supplementation significantly elevated Ace and Chao-1 indices of ruminal microbiota (*p* < 0.05). Differential ruminal metabolites were predominantly enriched in pathways involving pantothenate and CoA biosynthesis, regulation of lipolysis in adipocytes, thermogenesis, and lysine degradation. *Prevotellaceae_UCG-001* was negatively correlated with average daily feed intake (ADFI) and immunoglobulin M (IgM) (*p* < 0.05), and ascorbate radical and ureidoperacrylic acid showed significant correlations with ADFI, IgM, and *Prevotellaceae_UCG-001* (*p* < 0.05). Both *Anaerobispirillum* and *Quinella* were negatively correlated with TNF-α (*p* < 0.05), whereas thiamine and zeatin showed positive correlations with TNF-α and *Quinella* (*p* < 0.05). Under the present experimental conditions, the optimal supplemental level of YSE was 400 mg/kg diet.

## Simple Summary

Excessive use of antibiotics in large-scale goat fattening causes drug residues in goat products and impedes the green and sustainable development of goat farming, highlighting an urgent demand for safe and effective alternatives to antibiotics. *Yucca schidigera* extract (YSE) is a natural active substance obtained from yucca plants with antioxidant and anti-inflammatory benefits. Dietary supplementation with YSE in fattening goats improved animal growth performance, enhanced serum immunity, and stabilized the rumen microbiome, suggesting that YSE has promising potential as a widely used feed additive for goat fattening.

## 1. Introduction

In commercial livestock production, there is still the practice of adding antibiotics to the feed to alleviate production pressure [1,2,3], maintain animal health status [4,5], and promote growth performance [6,7]. Nevertheless, excessive antibiotic administration leads to prominent issues such as livestock product drug residues and antibiotic resistance, which severely restricts the sustainable and green development of the animal husbandry industry [8,9]. Accordingly, developing safe and efficacious plant-derived feed additives to substitute conventional antibiotic growth promoters carries important theoretical significance and practical application value for advancing healthy livestock production [6,10].

The *Yucca schidigera* extract (YSE) is a powdered bioactive substance extracted from the roots, stems, leaves, flowers, and bark of Yucca plants. The primary bioactive components of YSE are saponins, polyphenols, and polysaccharides with antioxidant, anti-inflammatory, and antiviral activities [11]. Saponins in YSE can synergize with resveratrol to exert anti-inflammatory effects [12,13], and the phenolic compounds in YSE exhibit strong free-radical-scavenging activity and are capable of inhibiting intracellular free-radical production [14,15]. In livestock, YSE improves antioxidant enzyme activities, immune status and hepatic antioxidative metabolism, as reported in dairy calves and fattening lambs [16,17]. Growing evidence from ruminant studies has confirmed that YSE modulates ruminal fermentation: it stabilizes ruminal pH, decreases rumen ammonia-nitrogen, optimizes volatile-fatty-acid profiles and reshapes rumen microbiota, further benefiting growth performance and product quality in cattle and lambs [18,19,20,21,22]. Snelling et al. [23] also found that YSE buffered rumen pH fluctuation and linearly increased milk fat content in dairy cows. Collectively, previous studies indicate that YSE exerts positive effects on livestock immunity, microbial homeostasis, and production performance. Nevertheless, relevant research on fattening goats remains limited, and the mechanisms by which YSE regulates rumen microbiota, metabolic profiles, growth performance, and immune functions in fattening goats are still poorly understood, which deserves further investigation.

Therefore, in this study, Boer goats were selected as the experimental animals to investigate the influences of the YSE on growth performance, serum immune parameters, rumen microbiota, and metabolome, so as to lay a theoretical foundation for the scientific and rational application of YSE in goat breeding production.

## 2. Materials and Methods

### 2.1. Preparation of Yucca schidigera Extract

The YSE used in the experiment was a commercial product purchased from Xi’an Baolekang Animal Health Co., Ltd. (Xi’an, China), with a natural steroid saponin content of ≥10%, and the remaining constituents were natural yucca-derived compounds, mainly polysaccharides, flavonoids, crude fiber, moisture, and other secondary metabolites. The extract was derived from the stems and leaves of the yucca plant.

### 2.2. Animals, Feed, and Experimental Design

The whole feeding experiment was conducted from April to May 2024 at the Zhengding Experimental Base of the Shijiazhuang Academy of Agricultural and Forestry Sciences. During the experimental period, the average ambient temperature was 21.0 °C, and the relative humidity ranged from 42% to 68%. Thirty-two healthy male Boer goats aged approximately 5 months with similar initial body weights (36.91 ± 3.05 kg) were selected for this trial. A single-factor randomized block design was adopted to allocate experimental animals into different groups; the average initial body weight showed no significant intergroup differences, which eliminated weight bias for subsequent measurements. Thirty-two goats were randomly divided into 4 groups with 8 replicates per group. Before the formal trial commenced, all goats underwent a 7-day pretrial adaptation period, followed by a 35-day official feeding trial. Four dietary treatments were set as follows: Control group (Group A) fed basal diet only; the low-dose group (Group B, basal diet supplemented with 200 mg/kg YSE; the medium-dose group (Group C, basal diet supplemented with 400 mg/kg YSE); and the high-dose group (Group D, basal diet supplemented with 600 mg/kg YSE). The basal diet was provided by a commercial feed enterprise, and nutrient levels complied with the nutritional requirements specified by the National Research Council (NRC, 2007) [24]. Corresponding amounts of YSE were accurately weighed and fully blended into the basal diet for each treatment group. The mixed feed was then extruded into cylindrical pellet feeds (diameter: 0.5 cm; length: 1.5–2.5 cm) to prepare total mixed rations (TMR) for ad libitum feeding. The ingredient composition and nutritional values of the basal diet are presented in Table 1.

### 2.3. Animal Management

All pens were equipped with slatted bamboo flooring to allow feces and urine to drain and accumulate on the floor surface. Prior to trial initiation, all goat pens were thoroughly cleaned and disinfected, and each experimental goat was tagged with a unique numbered ear tag for individual identification and data recording. Throughout the trial, all goats were individually housed in separate pens within a single barn under a fixed feeding regimen; diets were offered at 07:00 and 17:00 daily. Daily feed allotments were precisely recorded to ensure continuous feed availability in troughs; residual feed was collected and weighed on the following morning to calculate average daily feed intake (ADFI). Goats had free access to both feed and clean drinking water. Before and after the experimental period, all goats were fasted overnight and subsequently weighed to calculate the average daily gain (ADG) and feed-to-gain ratio (F/G). Staff members regularly observed their feeding and drinking behaviors, as well as clinical symptoms including diarrhea, fever and abnormal activity, with all abnormal conditions documented in a timely manner. Pens and water troughs were cleaned routinely during the entire experimental period, and cross-pen movement of goats was prohibited. Before the experiment began, in accordance with the livestock breeding standards and procedures, the routine deworming and immunization programs for the livestock herds were strictly carried out.

### 2.4. Sample Collection

On the morning of day 35 before feeding, four goats with body weight close to the group average were randomly selected from each treatment group for blood sample collection. Approximately 5 mL of venous blood was collected from the jugular vein using a 10 mL disposable syringe and transferred into serum coagulation tubes, which were clearly labeled individually. The blood samples were kept stationary at room temperature for 20 min, followed by centrifugation at 5000 rpm for 15–20 min. The obtained serum supernatant was dispensed into aliquots and preserved at −20 °C until subsequent serum biochemical and immune index detection.

Three hours after morning feeding on day 35, rumen fluid was harvested from the four preselected goats per group via an oral gastric tube rumen sampler (Wuhan Anscitech Animal Husbandry Technology Co., Ltd., Wuhan, China). The crude rumen contents were filtered through four layers of sterile gauze, and the filtrate was immediately divided into two portions in sterile cryogenic vials. All aliquots were snap-frozen in liquid nitrogen rapidly, then transferred and stored at −80 °C for subsequent rumen microbiota and metabolome sequencing analyses.

### 2.5. Analysis of Serum Inflammatory Cytokines and Immune Markers

Serum levels of inflammatory cytokines including IL-1β (Cat. No. H002-1-2), IL-6 (Cat. No. H007-1-2), and TNF-α (Cat. No. H019-1-2), as well as immunoglobulin markers (IgG, Cat. No. H107-1-2; IgA, Cat. No. H108-2-2; IgM, Cat. No. H109-1-2) were detected strictly in accordance with the operating instructions of commercial ELISA kits purchased from Nanjing Jiancheng Bioengineering Institute (Nanjing, China).

### 2.6. Analysis of the Rumen Microbiota Based on 16S rRNA Sequencing

Frozen rumen fluid samples were taken out from the −80 °C refrigerator and thawed on ice prior to total genomic DNA extraction. Microbial genomic DNA was extracted using the HiPure fecal DNA Kit (Cat. No. K210022, Thermo Fisher Scientific, Waltham, MA, USA), and DNA concentration and purity were quantified via the dsDNA HS Assay Kit (Cat. No. Q32851, Thermo Fisher Scientific, USA). Using 20–50 ng qualified genomic DNA as the template, the V3–V4 hypervariable regions of prokaryotic 16S rRNA gene were amplified by PCR. The primer sequences were as follows: forward primer 338F: 5′-ACTCCTACGGGAGGCAGCAG-3′; reverse primer 806R: 5′-GGACTACHVGGGTWTCTAAT-3′. Index adaptors were ligated to both terminals of PCR amplicons to construct sequencing libraries for next-generation sequencing (NGS).

After library concentration was verified using a spectrophotometer, paired-end 250 bp (PE250) sequencing was performed on the Illumina MiSeq 9 platform following the official instrument guidelines to acquire raw sequencing reads. Raw paired-end reads were assembled, and sequences containing ambiguous bases (N) and reads shorter than 200 bp were discarded. After quality filtering and chimera removal, the valid clean sequences were clustered into operational taxonomic units (OTUs) at a 97% sequence similarity threshold using VSEARCH software (v1.9.6) [25]. Taxonomic annotation of representative OTU sequences was conducted against the Silva138 reference database using the Bayesian algorithm embedded in the RDP classifier, and microbial community composition was summarized at multiple taxonomic ranks [26].

Based on OTU profiling results, α-diversity metrics (Shannon index, Chao1 index) were calculated through random subsampling to evaluate the richness and diversity of intrasample rumen microbial communities. Furthermore, weighted and unweighted UniFrac distance analyses were carried out to assess intergroup differences in overall microbial community structure across all samples.

### 2.7. Metabolomic Analysis of Rumen Fluid Using Liquid Chromatography–Mass Spectrometry

Frozen rumen fluid samples were thawed at 4 °C and vortexed for 1 min to achieve thorough homogenization. An appropriate volume of each sample was transferred into a 2 mL centrifuge tube, followed by the addition of 400 μL methanol solution and another 1 min of vortex mixing. The mixture was centrifuged at 12,000 rpm and 4 °C for 10 min; the resulting supernatant was collected into a new 2 mL tube and concentrated under vacuum until completely dry. The dried residues were reconstituted with 150 μL 4 ppm 2-chloro-L-phenylalanine internal standard solution (solvent: 80% aqueous methanol). The reconstituted solution was filtered through a 0.22 μm microporous membrane filter, and the filtrate was transferred into sample vials for subsequent ultra-high-performance liquid chromatography-mass spectrometry (UHPLC-MS) detection.

All samples were analyzed on an UHPLC-MS platform manufactured by Thermo Fisher Scientific (USA). Raw metabolomic data were preprocessed using the R-based XCMS package (Version 3.22.0) for peak picking, peak filtering, and peak alignment to generate a quantitative metabolite feature table [27]. Support vector regression (SVR) normalization was performed based on quality control (QC) samples. Only metabolites with a coefficient of variation (CV) less than 30% across all QC samples were retained for subsequent statistical verification and differential metabolite analysis. Primary-level differential rumen metabolites were screened with variable importance in projection (VIP) > 1.0 and *p* < 0.05, without FDR correction or fold-change filtering.

### 2.8. Statistical Analysis

All experimental data were statistically analyzed using IBM SPSS Statistics 27.0 software (IBM Corporation, Armonk, NY, USA). One-way analysis of variance (one-way ANOVA) was adopted to compare differences among treatment groups. Levene’s test was performed prior to ANOVA to verify the homogeneity of variance across groups. Upon obtaining significant overall differences via ANOVA, Duncan’s multiple range test was applied for post hoc multiple comparisons between groups. To systematically elucidate the intrinsic regulatory pathways by which the YSE regulates the growth and immune performance of fattening goats. The primary-level metabolites were further subjected to secondary filtering with stricter criteria: VIP > 1, FDR < 0.05, and fold change (FC) > 2 or FC < 0.5; and conducted a correlation analysis of the selected rumen metabolites, rumen microbiota, growth performance indicators, and serum immune indicators. Pearson correlation analysis was conducted with SPSS 27.0, and correlation heatmaps were visualized using GraphPad Prism 8.0. The *p* < 0.05 was defined as the threshold for statistically significant difference, and values of 0.05 ≤ *p* < 0.10 were regarded as statistical trends with potential biological implications and were documented accordingly.

## 3. Results

### 3.1. Growth Performance

Table 2 presents the effects of YSE on growth performance in fattening goats. Compared with Group A, the ADG in Group D was higher by 38.07% (*p* < 0.05). Conversely, the F/G in Group D was lower by 32.41% (*p* < 0.05) than that in Group A. Similarly, the F/G in Group D was lower compared with Group C (*p* < 0.05), and Group B had no significant difference compared with all other groups (*p* > 0.05). No significant differences were observed in the remaining measured parameters among the four groups (*p* > 0.05).

### 3.2. Serum Immunological Markers

Table 3 presents the effects of YSE on serum immune parameters in fattening goats. Compared with Group A, serum IgM levels in Group C increased significantly by 60.57% (*p* < 0.05). Conversely, serum TNF-α levels in Group C were significantly lower by 38.98% compared with Group A (*p* < 0.05). The addition of YSE to the feed had no significant effect on the concentrations of serum IgA, IgG, IL-1β, and IL-6 (*p* > 0.05).

### 3.3. Rumen Microbiota

The rarefaction curve acquired by high-throughput 16S rRNA gene sequencing is illustrated in Figure 1A. The initial phase demonstrates a positive correlation between sequencing depth and the number of operational taxonomic units (OTUs) identified. The curve subsequently enters a plateau phase, indicating that a further increase in the volume of sampled data does not lead to an increase in the number of detected OTUs. This phenomenon suggests that the volume of sequencing data employed in this study is sufficient to meet the analytical requirements. As shown in Figure 1C, OTU clustering analysis and subsequent Venn diagram construction were used to thoroughly analyze the OTUs common to all groups (including control Group A and experimental groups B, C, and D) and those specific to each group. The results show that groups A, B, C, and D contained 1672, 1695, 1816, and 1632 OTUs, respectively. It is worth noting that 1359 core OTUs were shared across all four groups. Furthermore, the number of OTUs specific to groups A, B, C, and D was 22, 22, 63, and 39, respectively. These findings strongly demonstrate significant differences in the rumen microbial communities between the different groups. The principal coordinate analysis graph shows that there is sample overlap among groups A, B, and D. Group C is closely clustered, with relatively small differences within the group, and most of the samples are close to each other. The community composition is relatively similar. Specifically, principal coordinate 1 and principal coordinate 2 explained 17.1% and 12.65% of the variation, respectively (Figure 1B). ANOSIM is a non-parametric test used to determine whether the differences between groups are significantly greater than the differences within groups, thereby assessing the significance of the grouping. Based on the ANOSIM analysis (R = 0.061; *p* = 0.21), there was no significant difference in the Beta diversity of rumen microbial communities among the groups, indicating that adding different doses of yucca extract did not have a significant impact on the rumen microbial community structure of fattening goats (Figure S1).

As shown in Figure 1D, the Chao1 index and ACE index in Group C were higher than those in Group D (*p* < 0.05). Although there were no significant differences in these two indices between Groups B, C, and D compared with Group A (*p* > 0.05). However, Group C showed an upward trend, whereas Group D showed a downward trend. There were no significant differences in the Shannon index or Simpson’s index among the four groups (*p* > 0.05). Coverage exceeded 99% in all groups, indicating that the sequenced samples adequately represented the microbial community characteristics of the rumen of fattening goats, thereby accurately depicting the bacterial community composition of all samples.

At the phylum level, the distribution of species is shown in Figure 2A. The four most abundant phyla detected across all treatments were Bacteroidota, Firmicutes, Proteobacteria, and Spirochaetes. Among them, Bacteroidota and Firmicutes were overwhelmingly predominant, accounting for 93.60%, 92.75%, 89.57%, and 93.68% of the total abundance in each group, respectively. Proteobacteria and Spirochaetes were also present in all groups but exhibited much lower relative abundance than Bacteroidota and Firmicutes.

At the family level, the Prevotellaceae and Bacteroidaceae were the dominant bacterial families, accounting for 54.77%, 46.58%, 38.52%, and 49.45% of the total abundance in each group, respectively. It is worth noting that the relative abundance of the Prevotellaceae in Group C was significantly lower than that in Group A (*p* < 0.05), and showed a downward trend compared to Groups B and D (Figure 2B). Further classification of the bacterial communities within the samples revealed the distribution of relative abundances at the genus level, as shown in Figure 2C. The dominant bacterial taxa included *Prevotella*, *Rikenellaceae_RC9_gut_group*, *Bacteroidales_RF16_group*, and *NK4A214_group*. Among them, the relative abundance of *Prevotella* and *Bacteroidales_RF16_group* varies in the four groups. The relative abundance of *Prevotella* in Group C was lower than that in Group A (*p* < 0.05), showing a decreasing trend compared to Groups B and D. The relative abundance of *Bacteroidales_RF16_group* in Group C was higher than that in Group A (*p* < 0.05), showing an increasing trend compared to Groups B and D. Additionally, the relative abundance of *Prevotellaceae_UCG-001* in Group D was higher than that in the other groups (*p* < 0.05).

Using STAMP software (v2.1.3) [28], significant differences in bacterial species abundance were compared across different taxonomic levels, with a focus on analyzing species abundance differences at the genus level. As shown in Figure 2D, a total of nine bacterial genera (*Anaerobiospirillum*, *Desulfovibrio*, *Fibrobacter*, *Lachnospiraceae_XPB1014_group*, *Neisseria*, *Prevotellaceae_UCG-001*, *Quinella*, *VadinBE97*, and *WCHB1-41*) exhibited significant differences between the different groups.

### 3.4. Rumen Metabolites

Untargeted metabolomics was employed to characterize ruminal metabolites, and principal component analysis (PCA) was performed for dataset classification to holistically visualize the overall structure of metabolic profiles. This step helps to exclude outlier samples, thereby improving model accuracy. As shown in Figure 3A,B, the distribution points for each sample group are relatively clustered, indicating that these samples are similar in composition and concentration. Different groups are denoted by different colors, and 95% confidence intervals are plotted, indicating a degree of separation between the groups. Furthermore, the cross-validation R^2^X parameters of the reference models (positive ion mode: 0.520; negative ion mode: 0.528) all exceed 0.5, indicating that the models possess good interpretability. The use of OPLS-DA analysis further reduces model complexity and enhances its interpretability, thereby maximizing the presentation of inter-group differences. As shown in Figure 3C,D, samples from different groups are dispersed along the horizontal axis, whilst samples within groups cluster along the vertical axis, fully demonstrating the reproducibility of the samples and the reliability of the results.

To achieve accurate classification of the detected metabolites, secondary qualitative identification was performed by searching and comparing spectral databases such as the Human Metabolome Database (HMDB), the Lipid Atlas, the Kyoto Encyclopedia of Genes and Genomes (KEGG), and our in-house metabolite reference database. As shown in Figure 4, compared with Group A, Group B exhibited 54 significantly up-regulated metabolites and 55 significantly down-regulated metabolites; Group C showed 174 significantly up-regulated metabolites and 87 significantly down-regulated metabolites; whilst Group D exhibited 76 significantly up-regulated metabolites and 94 significantly down-regulated metabolites.

By performing KEGG pathway enrichment analysis on the list of differentially expressed metabolites, we aimed to explore the functional mechanisms underlying these metabolic differences. The results indicate that the differentially expressed metabolites between Group A and Group B are primarily enriched in pathways such as pantothenic acid and coenzyme A biosynthesis, regulation of lipolysis in adipocytes, thermogenesis, breast cancer, and chemical carcinogenesis—receptor activation (Figure 5A). The differentially expressed metabolites between Group A and Group C were primarily enriched in pathways such as lysine degradation, TGF-β signaling, gap junctions, phytohormone signal transduction, and the synaptic vesicle cycle (Figure 5B). Furthermore, the differentially expressed metabolites between Group A and Group D were primarily enriched in pathways related to prostate cancer, cancer, breast cancer, caffeine metabolism, and thermogenesis (Figure 5C).

### 3.5. Correlation Analysis

Correlations between rumen bacterial genera, growth performance, and immune indicators are illustrated in Figure 6A. Both ADFI and IgM were negatively correlated with *Prevotellaceae_UCG-001* (*p* < 0.05). TNF-α exhibited an extremely significant positive correlation with *Neisseria* (*p* < 0.01) and a significant negative correlation with *Anaerobispirillum* and *Quinella* (*p* < 0.05).

The correlation between growth performance, immune indicators, and rumen metabolites is shown in Figure 6B. ADFI showed an extremely significant negative correlation with ureidoperacrylic acid (*p* < 0.01). Additionally, ADFI was significantly negatively correlated with ascorbate radical, dicyclohexylamine, epilubimin, and lysylhydroxyproline (*p* < 0.05), while it had significant positive correlations with 6-hydroxymethyl-7,8-dihydroterpin and patuletin (*p* < 0.05). IgM was extremely significantly negatively correlated with dicyclohexylamine and ureidoperacrylic acid (*p* < 0.01), and significantly negatively correlated with 2-aminoheptanedioic acid, ascorbate radical, D-myo-inositol 3-phosphate, and sinapine (*p* < 0.05). Moreover, IgM was positively associated with 6-hydroxymethyl-7,8-dihydroterpin (*p* < 0.05). TNF-α was positively correlated with FAL 16_1 (*p* < 0.05) and negatively correlated with thiamine and zeatin (*p* < 0.05).

The correlation analysis between rumen bacteria genera and metabolites is shown in Figure 6C. *Prevotellaceae_UCG-001* showed extremely significant positive correlations with (1R,4S,5R)-4-thujanol, ascorbate radical, D-myo-inositol 3-phosphate, dicyclohexylamine, and ureidoperacrylic acid (*p* < 0.01). Meanwhile, *Prevotellaceae_UCG-001* was also positively correlated with 2-aminoheptanedioic acid and sinapine (*p* < 0.05), and negatively correlated with 6-hydroxymethyl-7,8-dihydroterpin (*p* < 0.05). *Anaerobispirillum* was negatively correlated with epilubimin, FAL 16_1, lysylhydroxyproline, and sebacic acid (*p* < 0.05). *Quinella* exhibited extremely significant positive correlations with 3-carboxy-4-methyl-5-propyl-2-furanpropionic acid, patuletin, and gamma-glutamylleucine (*p* < 0.01), as well as significant positive correlations with paspalinine and thiamine (*p* < 0.05). Meanwhile, *Quinella* had an extremely significant negative correlation with sebacic acid and zeatin (*p* < 0.01), and was positively correlated with 3-(3-hydroxyphenyl) propanoic acid, 5C-aglycone, epilubimin, lysylhydroxyproline, and umbelliferone (*p* < 0.05).

## 4. Discussion

The ADG and F/G are routinely adopted as core evaluation indices to assess the growth performance of livestock in feeding trials. Johnson et al. [29] reported that the addition of yucca extract significantly affected the rumen microbial community in ruminants and the production performance of dairy cows. In particular, it reduced the urea nitrogen in whey and rumen ammonia content, thereby improving nitrogen utilization efficiency and enhancing the overall performance of the animals. In this experiment, we found that the addition of high doses of YSE can promote growth by improving feed utilization. The ADG in Group D was significantly higher than in the other three groups, and the F/G was significantly lower than in Groups A and C. This is largely consistent with the findings of Soroor et al. [30], who reported that plant extract supplementation improved the growth rate of Mehraban lambs. Meanwhile, Ahmed et al. [31] reviewed that phytobiotic additives such as YSE can improve the growth performance of ruminants while reducing nitrogen excretion and methane emissions.

In addition to improving production performance, YSE has also demonstrated good immunomodulatory potential. Group C exhibited higher serum IgM levels and lower TNF-α levels. IgM acts as the body’s ‘first line of defense’ in the humoral immune response against foreign invaders, whilst the downregulation of the pro-inflammatory factor TNF-α suggests that YSE can effectively enhance humoral immune function and alleviate inflammatory responses. This may be attributed to the high content of components such as steroidal saponins and resveratrol in the YSE, which can modulate cellular and humoral immunity, activate lymphocyte function, increase immunoglobulin production, and reduce the release of inflammatory cytokines such as NO and TNF-α by downregulating the expression of relevant mRNA genes [32,33,34].

It is worth noting that YSE can regulate the balance of the microbial flora in the animal gut through its active components, thereby enhancing the body’s immune function [35]. In the results of this experiment, the ACE and Chao1 indices in Group C were significantly elevated, indicating that this dosage substantially increased the species richness of the gut microbiota in fattening goats. A highly diverse gut microbiota implies greater efficiency in intestinal digestion and absorption, as well as enhanced disease resistance; it also provides a sound microecological foundation for optimizing the body’s immune function. This is logically consistent with the optimal immune status exhibited by Group C (high IgM, low TNF-α). Furthermore, significant differences were observed in nine bacterial genera across the groups, including *Anaerobiospirillum*, *Desulfovibrio*, *Fibrobacter*, *Lachnospiraceae_XPB1014_group*, *Neisseria*, *Prevotellaceae_UCG-001*, *Quinella*, *VadinBE97* and *WCHB1-41*, which is highly consistent with the results of pathway enrichment analysis of differentially expressed metabolites between groups in this study. Specifically, the differentially expressed metabolites in Group A compared with Groups B and D were primarily enriched in pathways such as pantothenic acid and coenzyme A biosynthesis, regulation of lipolysis in adipocytes, and thermogenesis. These pathways are closely related to lipid metabolism and energy conversion. This finding is highly consistent with our unpublished observations regarding increased crude fat digestibility and lipase activity, and a strong correlation can be inferred between these metabolic pathways and the phenotypic indicators. Similar plant-derived formulations have been reported to modulate gut microbiota and host antioxidant status [36], and dietary lipid interventions can reshape rumen microbial populations and nutrient digestion in ruminants [37]. This also reveals that high-dose yucca extract primarily promotes growth in fattening goats by regulating lipid metabolism and energy conversion processes. In contrast, the differentially expressed metabolites between Group A and Groups C were mainly enriched in pathways such as lysine degradation and the TGF-β signaling pathway. Santoso et al. [38] reported that plant-derived saponins can improve ruminal nitrogen utilization and microbial protein synthesis in goats, while Eryavuz et al. [39] showed that dietary YSE alters the concentration of rumen microorganisms in sheep.

Huang et al. [40] found that lysine can upregulate anti-inflammatory genes via the TOR/NF-κB signaling pathway and downregulate pro-inflammatory genes via the p38MAPK/NF-κB signaling pathway, thereby enhancing the intestinal immune function of grass carp fry. Mushtaq et al. [41] demonstrated that as digestible lysine levels increase, antibody titres against Newcastle disease and infectious bursal disease viruses rise significantly. Morikawa et al. [42] found that TGF-β exerts potent immunosuppressive effects by influencing cell differentiation and proliferation. Combining the central roles of lysine and TGF-β in immune regulation and cellular function further confirms, at the molecular level, the significant advantages of Group C in terms of immune function.

Correlation analysis revealed that ADFI was significantly negatively correlated with *Prevotellaceae_UCG-001*, and metabolites including ureidoperacrylic acid, ascorbate radical, dicyclohexylamine and 6-hydroxymethyl-7,8-dihydroterpin served as intermediate substances linking these two indicators. Previous studies had verified that *Prevotellaceae_UCG-001* could modulate the production of ureidoperacrylic acid and ascorbate radical, thereby regulating ADFI. Manlapig et al. [43] confirmed that elevated abundance of Prevotellaceae accelerated lactic acid synthesis in the rumen, which raised the level of the propanoate intermediate ureidoperacrylic acid and consequently boosts propionate production. Increased propionate suppresses feed intake in ruminants via the hypothalamus, ultimately reducing ADFI [44]. Li et al. [45] further reported that enrichment of Prevotellaceae upregulates the ascorbic acid metabolic pathway, which may eventually inhibit ADFI, consistent with previous insights regarding vitamin-C-related stress responses in ruminants [46]. Consistent with these findings, our study observed that both ureidoperacrylic acid and ascorbate radical exhibited extremely significant positive correlations with *Prevotellaceae_UCG-001*, as well as prominent negative correlations with ADFI. In addition, the present results indicated that these two metabolites were also closely associated with IgM and *Prevotellaceae_UCG-001*, showing significant negative correlations with IgM. Prevotellaceae stimulated cortisol synthesis by promoting ascorbate radical generation, and excessive cortisol accumulation triggered B lymphocyte apoptosis and impeded IgM synthesis, whereas exogenous vitamin C supplementation could eliminate ascorbate radical and relieved its inhibitory effect on IgM secretion [47]. Meanwhile, excess propionate derived from ureidoperacrylic acid induced by Prevotellaceae triggers metabolic stress in ruminants, which coordinately activated the HPA axis to increase cortisol levels and indirectly suppressed IgM production [48]. The present study revealed that both *Anaerobiospirillum* and *Quinella* were significantly negatively correlated with TNF-α. As a non-resident rumen genus belonging to the family Succinivibrionaceae, *Anaerobiospirillum* can utilize starch, glucose, and soluble oligosaccharides as carbon substrates. This bacterium fixes CO_2_ via phosphoenolpyruvate carboxykinase to massively synthesize succinate, thereby promoting microbial crude protein synthesis [49,50]. Physiological levels of succinate secreted by *Anaerobiospirillum* have been demonstrated to suppress the macrophage NF-κB signaling pathway, ultimately reducing the production and release of TNF-α [51]. Furthermore, the present study indicated that thiamine and zeatin serve as key metabolites linking *Quinella* and TNF-α, with both metabolites exhibiting significant negative correlations with TNF-α. Kumar et al. [52] reported that the *Quinella* genome encodes key enzymes responsible for de novo thiamine biosynthesis. Adequate thiamine facilitates glucose fermentation by *Quinella* to produce lactate and propionate, which reduces ruminal hydrogen partial pressure and decreases the accumulation of LPS and TNF-α. In addition, activated thiamine targets ruminal epithelial cells and mucosal macrophages, inhibiting the TLR4/NF-κB/p38 signaling cascade and thereby directly repressing the transcription and secretion of TNF-α [53]. As a plant-specific cytokinin, zeatin cannot be synthesized by rumen microorganisms and is primarily derived from dietary intake [54]. Lappas et al. [55] demonstrated, in an in vitro cellular study, that zeatin binds to the adenosine A2A receptor in mammalian immune cells, blocks the NF-κB signaling pathway, and attenuates TNF-α secretion by macrophages and lymphocytes. Correlation analysis revealed an extremely significant negative correlation between zeatin and *Quinella*. Therefore, further research is required to explore their utilization relationship, which can provide theoretical support for regulating ruminal zeatin content and alleviating inflammatory responses.

Nevertheless, in this study, the sample size for each group of the rumen microbiome and metabolome analysis was 4. Considering the significant individual differences in the rumen microbial communities of ruminants, this experiment still has some limitations. Further verification of the findings of this study is needed with larger sample sizes in subsequent experiments.

## 5. Conclusions

Dietary supplementation with *Yucca schidigera* extract (YSE) positively regulated growth performance, immune status, and rumen micro-metabolome of fattening goats. The 600 mg/kg YSE addition increased average daily gain and feed efficiency, whereas 400 mg/kg YSE improved humoral immunity, mitigated inflammation, and optimized rumen bacterial diversity and composition. Key taxa such as *Prevotellaceae_UCG-001*, *Anaerobispirillum*, and *Quinella* were closely associated with host growth, immune indices, and rumen metabolites, contributing to improved physiological status. Under conditions of this trial, 400 mg/kg is recommended as the optimal dietary YSE supplementation level for fattening goats.

## Figures and Tables

**Figure 1:**
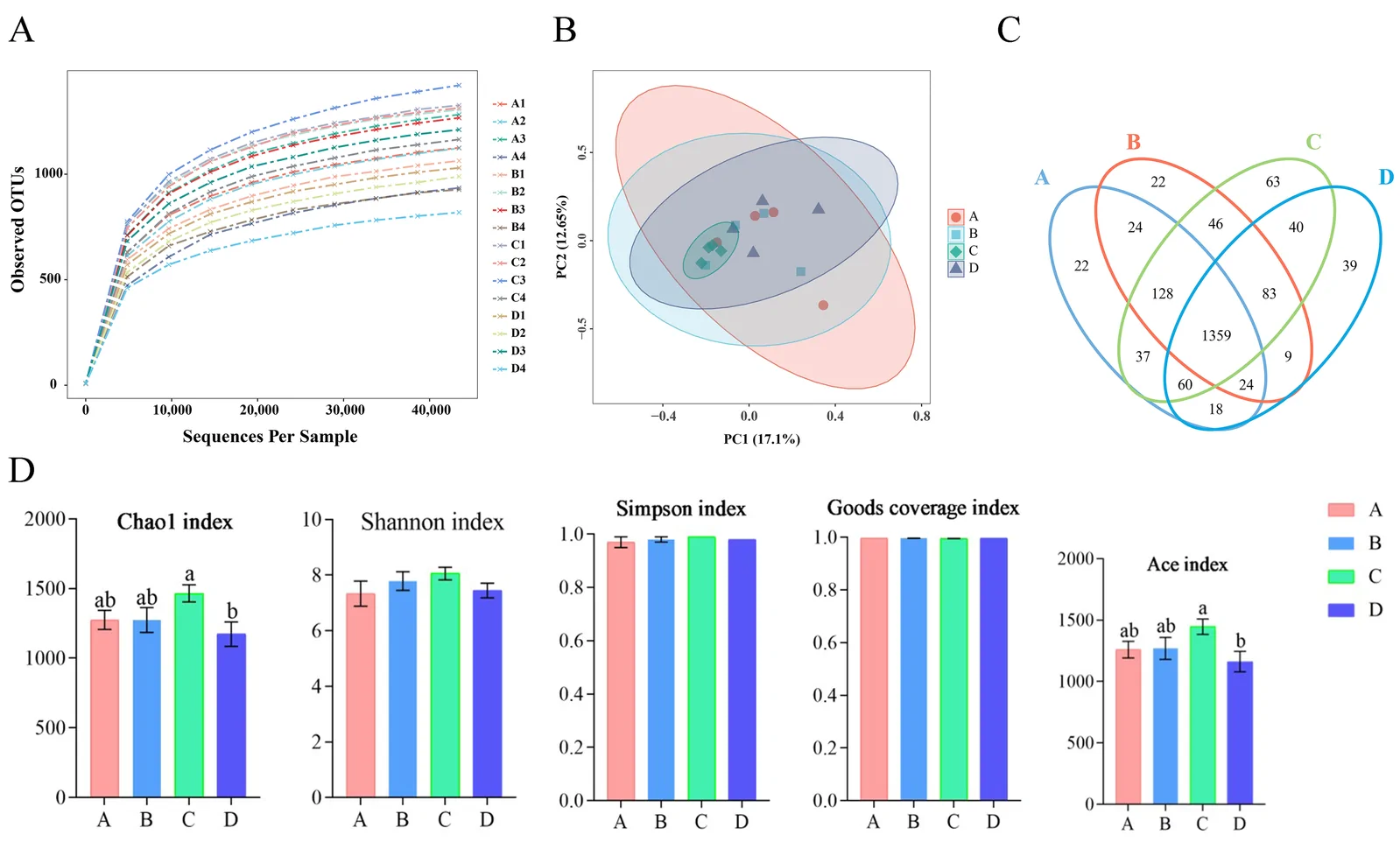
Effects of YSE on alpha diversity, beta diversity and OTU composition of rumen microbial communities in fattening goats. (**A**) Rarefaction curve; (**B**) PCoA analysis of rumen microbial beta diversity; (**C**) OTU Venn diagram; (**D**) Alpha diversity indices. Values with different lowercase letters are significantly different between treatments (*p* < 0.05).

**Figure 2:**
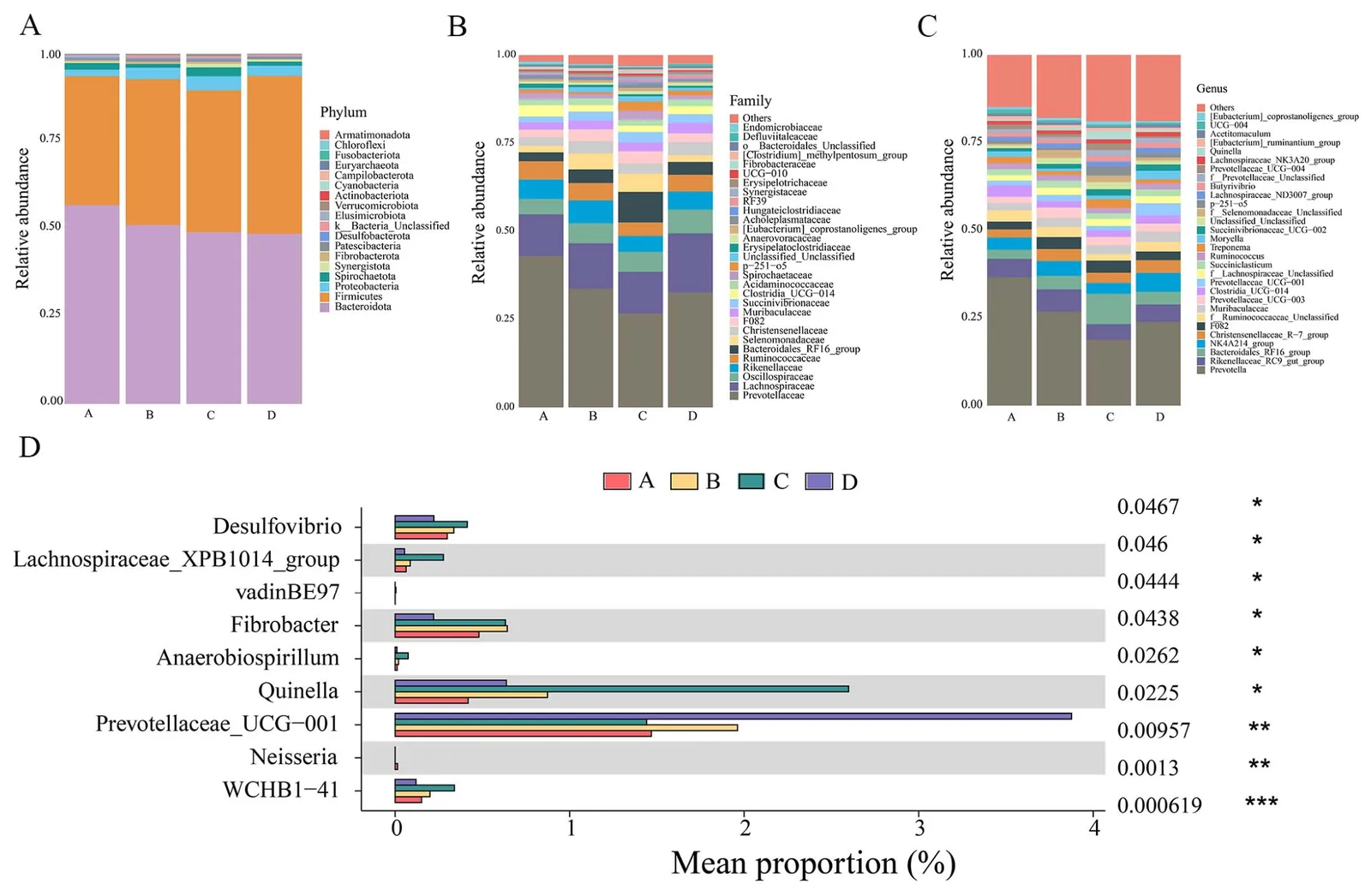
Rumen microbial community composition of four groups at phylum, family, and genus taxonomic levels. (**A**) Relative abundance bar chart of rumen microorganisms at the phylum level; (**B**) Relative abundance bar chart at the family level; (**C**) Relative abundance bar chart at the genus level; (**D**) Mean proportion and significant difference analysis of significantly differential genera among groups. Different colors represent different microbial taxa; (**A**–**D**) stand for four experimental groups; * indicates significant difference between groups (* *p* < 0.05, ** *p* < 0.01, *** *p* < 0.001).

**Figure 3:**
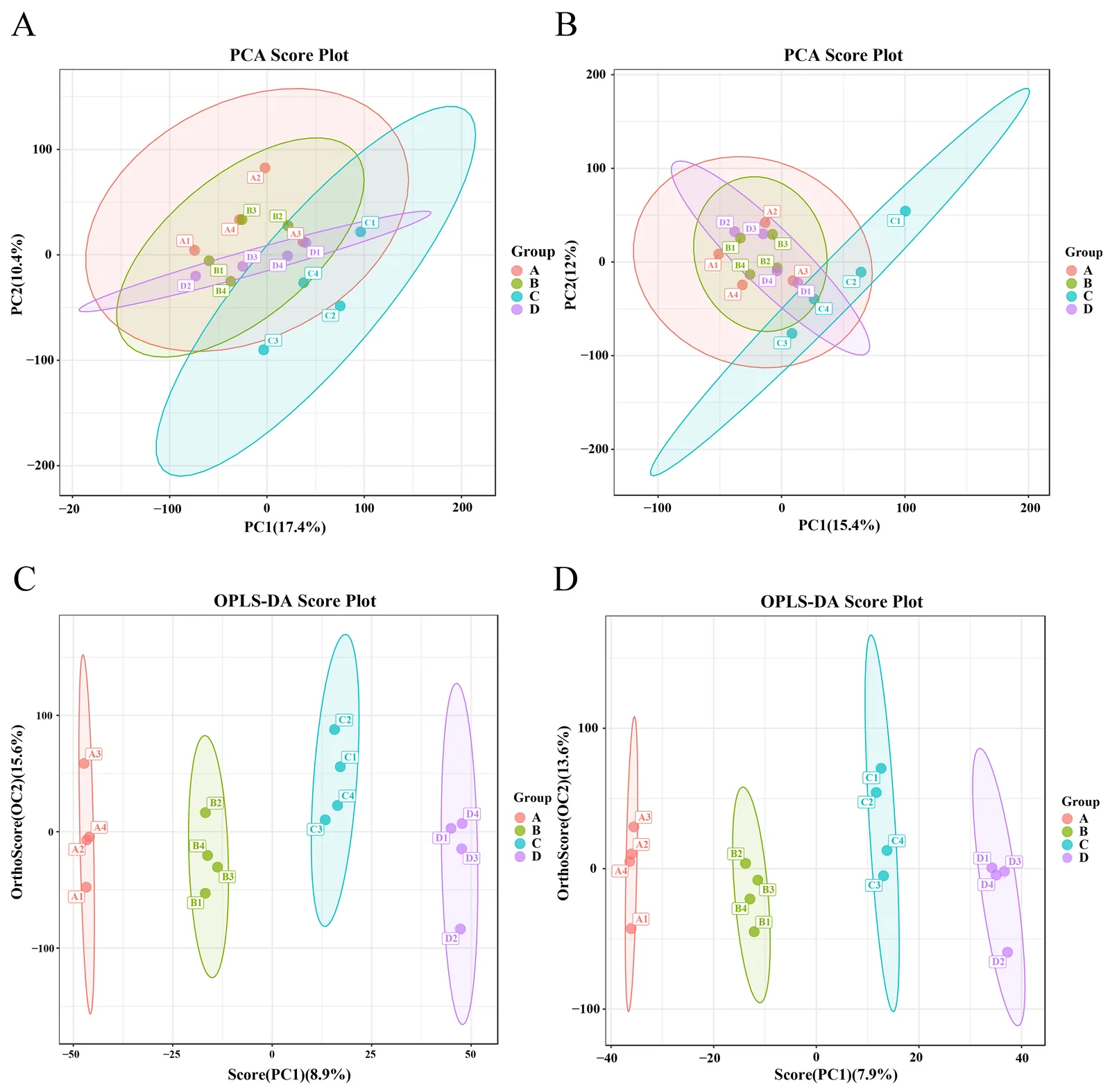
Multivariate statistical analysis score plots of metabolomics. (**A**) Principal Component Analysis (PCA) score plot in positive ion mode; (**B**) PCA score plot in negative ion mode; (**C**) Orthogonal Partial Least Squares Discriminant Analysis (OPLS-DA) score plot in positive ion mode; (**D**) OPLS-DA score plot in negative ion mode. Each dot represents a biological sample; different colors correspond to four experimental groups (**A**–**D**). The ellipse in the figure represents the 95% confidence interval of each group. The percentage on each axis indicates the proportion of total metabolic variation explained by the corresponding principal component.

**Figure 4:**
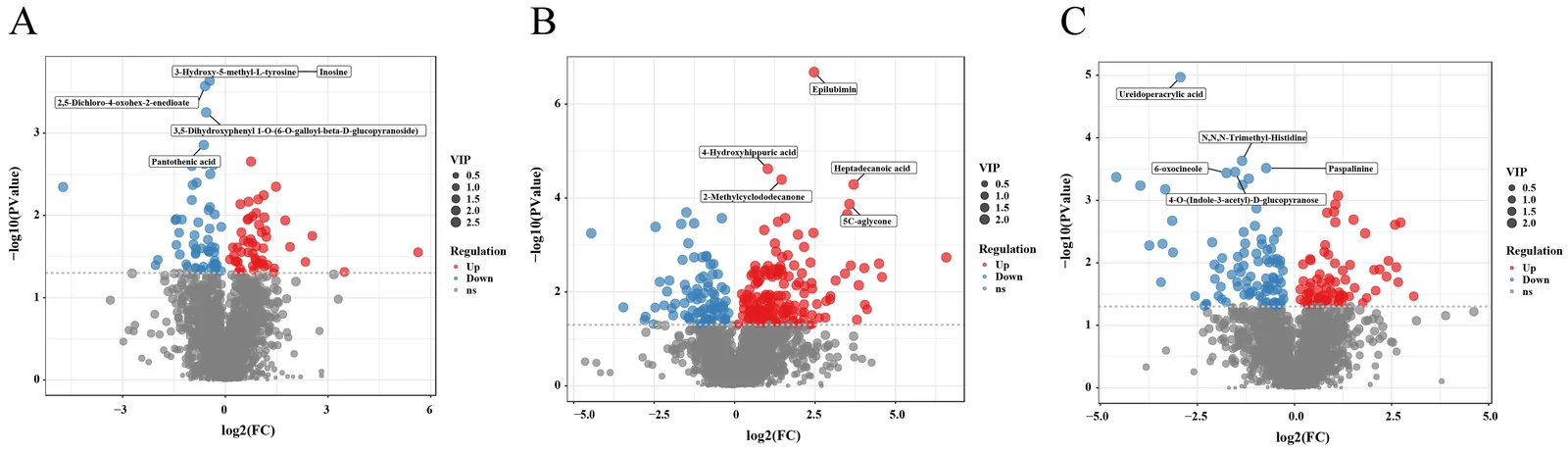
Comparison of secondary differential metabolites between different groups. (**A**) Volcano plot of differential metabolites between Group A and Group B; (**B**) Volcano plot of differential metabolites between Group A and Group C; (**C**) Volcano plot of differential metabolites between Group A and Group D. Red dots represent significantly up-regulated metabolites, blue dots represent significantly down-regulated metabolites, and gray dots indicate metabolites with no significant difference. The size of each dot corresponds to the variable importance in projection (VIP) value; larger dots denote higher VIP values.

**Figure 5:**
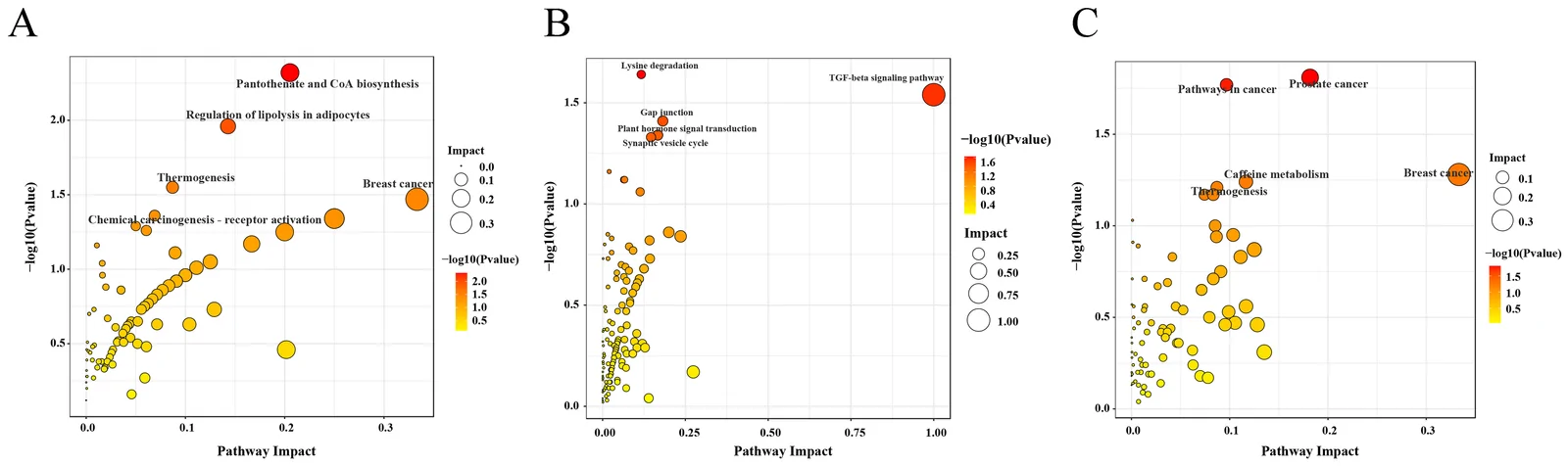
KEGG metabolic pathway enrichment analysis of differentially expressed metabolites across groups. (**A**) KEGG metabolic pathway enrichment analysis of differentially expressed metabolites between Group A and Group B. (**B**) KEGG metabolic pathway enrichment analysis of differentially expressed metabolites between Group A and Group C. (**C**) KEGG metabolic pathway enrichment analysis of differentially expressed metabolites between Group A and Group D. The *x*-axis represents the pathway impact score, and the *y*-axis shows −log10(*p*-value), with higher values indicating greater significance of pathway enrichment. The size of the dots corresponds to the pathway impact scores, while the color gradient from yellow to red reflects increasing enrichment significance.

**Figure 6:**
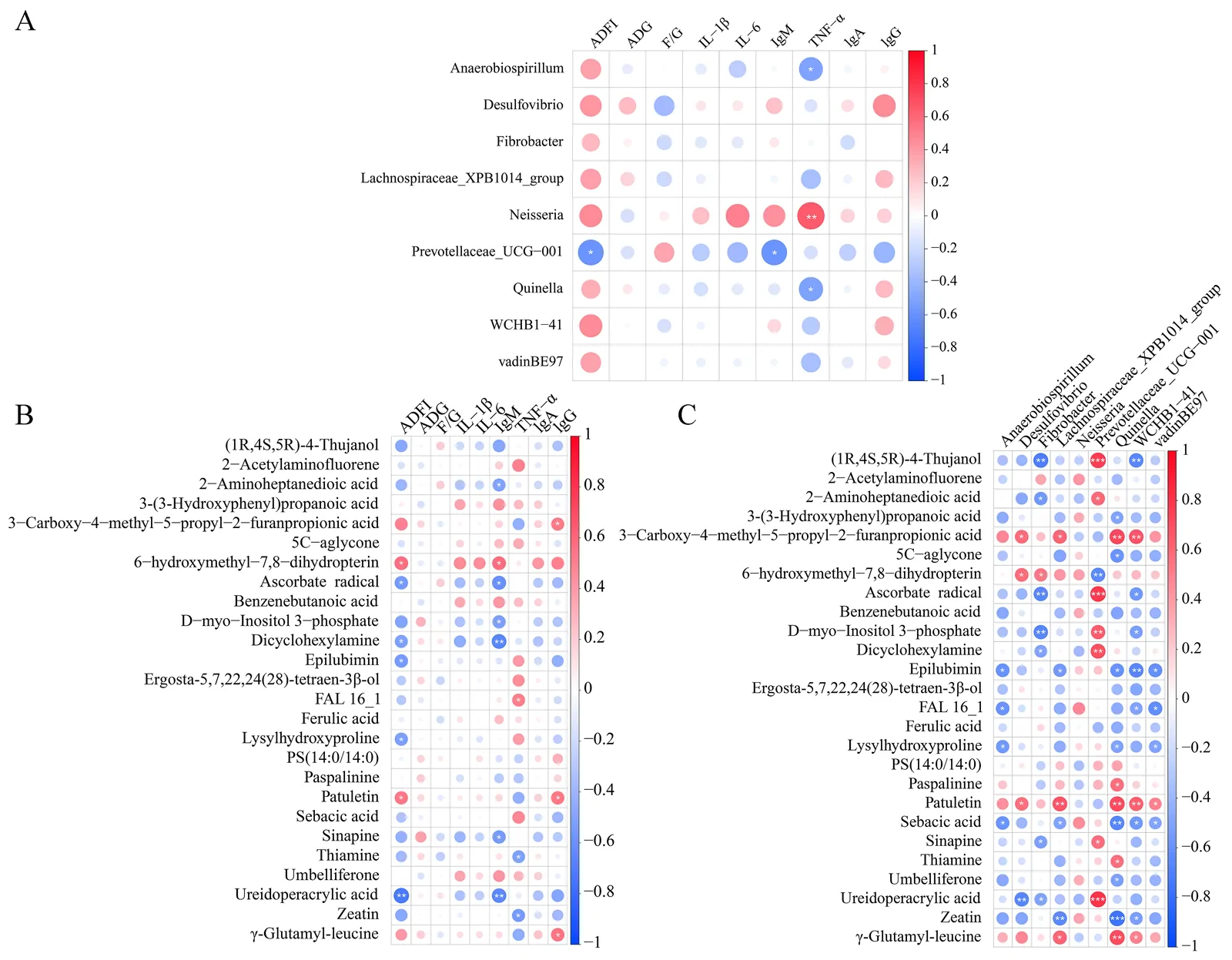
Correlation heatmaps among rumen microbiota, ruminal metabolites, growth performance, and serum immune indices of fattening goats. (**A**) Correlation heatmap between rumen microbiota, serum growth performance, and immune indices. (**B**) Correlation heatmap between ruminal metabolites, serum growth performance, and immune indices. (**C**) Correlation heatmap between rumen microbiota and ruminal metabolites. Red circles indicate positive correlations between variables, and blue circles represent negative correlations. The size of circles corresponds to the absolute value of correlation coefficient. * *p* < 0.05, ** *p* < 0.01, *** *p* < 0.001.

**Table 1:** Composition and nutrient composition of basic diet (air-dried basis, %).

Ingredient, %	Content	Nutrient Level	Measured
Corn	24.40	Digestible Energy, DE/(MJ/kg ^2^)	10.86
Soybean Meal	10.00	Dry Matter, DM	89.25
Corn Husk	6.00	Ether extract, EE	5.34
Wheat Bran	5.00	Crude Protein, CP	11.10
Peanut Vine Powder	52.00	Crude Ash, Ash	9.86
Baking Soda	0.40	Neutral detergent fiber, NDF	32.21
Dicalcium Phosphate	0.80	Acid detergent fiber, ADF	28.82
NaCl	0.40	Calcium, Ca	1.37
Premix ^1^	1.00	Phosphorus, P	0.79
Total	100		

^1^ The premix provided the following nutrients per kilogram of the basal diet: 10,000 IU of vitamin A, 600 IU of vitamin D_3_, 40 IU of vitamin E, 10 mg of copper (from copper sulfate pentahydrate), 70 mg of iron (from ferrous sulfate monohydrate), 60 mg of zinc (from zinc sulfate monohydrate), 60 mg of manganese (from manganese sulfate monohydrate), 1 mg of iodine (from potassium iodide), and 0.4 mg of selenium (from sodium selenite). ^2^ DE was calculated based on the proportion of each feed ingredient and the Nutrient Requirements of Domestic Animals (NRC, 2007), while the contents of other nutrients were measured values.

**Table 2:** Effects of YSE on growth performance of finishing goats.

Item	Group			
	A	B	C	D
Initial weight, kg	36.95 ± 6.47	37.05 ± 4.79	36.95 ± 4.59	39.87 ± 5.91
Final weight, kg	45.40 ± 6.07	46.05 ± 5.33	45.98 ± 5.27	51.53 ± 5.77
ADG, g/d	256.06 ± 18.74 ^b^	272.73 ± 20.02 ^b^	273.49 ± 30.36 ^b^	353.54 ± 5.35 ^a^
ADFI, g/d	1823.83 ± 59.34	1731.69 ± 58.20	1822.68 ± 64.66	1726.15 ± 69.87
F/G	7.22 ± 0.46 ^a^	6.44 ± 0.43 ^ab^	6.94 ± 0.83 ^a^	4.88 ± 0.07 ^b^

Note: Group A = control (basal diet); Group B = 200 mg/kg YSE in basal diet; Group C = 400 mg/kg YSE basal diet Group D = 600 mg/kg YSE basal diet. YSE, *Yucca schidigera* extract. Different lowercase letters indicate significant differences (*p* < 0.05).

**Table 3:** Effects of YSE on serum immune parameters of finishing goats.

Item	Group			
	A	B	C	D
IgA (mg/mL)	2.16 ± 0.21	1.76 ± 0.21	2.00 ± 0.18	1.80 ± 0.18
IgG (mg/mL)	10.05 ± 0.90	8.77 ± 0.90	10.94 ± 1.05	8.48 ± 0.80
IgM (mg/mL)	1.75 ± 0.25 ^b^	2.09 ± 0.22 ^b^	2.81 ± 0.22 ^a^	2.36 ± 0.16 ^ab^
IL-1β (ng/L)	22.65 ± 2.17	17.82 ± 1.69	19.64 ± 1.33	17.83 ± 1.94
IL-6 (ng/L)	103.91 ± 6.18	82.95 ± 8.87	90.76 ± 8.10	88.47 ± 11.24
TNF-α (ng/L)	110.81 ± 14.87 ^a^	86.85 ± 12.36 ^ab^	67.64 ± 3.49 ^b^	93.95 ± 10.21 ^ab^

Note: Group A = control (basal diet); Group B = 200 mg/kg YSE in basal diet; Group C = 400 mg/kg YSE basal diet; Group D = 600 mg/kg YSE basal diet. YSE, *Yucca schidigera* extract. Different lowercase letters indicate significant differences (*p* < 0.05).

## Data Availability

The datasets used during the current study are available from the corresponding authors on reasonable request. The data are not publicly available due to ethical restrictions regarding animal experimental data.

## Supplementary Materials

The following supporting information can be downloaded at: https://www.mdpi.com/article/10.3390/ani16193020/s1, Figure S1: ANOSIM inter-group difference analysis. Box-and-whisker plot illustrating intergroup differences based on ANOSIM. R = 0.061 and *P* = 0.21 represent the ANOSIM R-statistic and significance value, respectively. Boxes indicate the interquartile range, the black horizontal line within each box is the median, and whiskers show the minimum and maximum values of the dataset.
