# Supplementary material for: Rumen Microbiota and Metabolome Reveal the Effects of Yucca schidigera Extract on Growth Performance and Serum Immune Parameters in Fattening Goats
Source: Animals (Basel). 2026 Sep 25;16(19):3020. doi: 10.3390/ani16193020 (PMC13663501; doi:10.3390/ani16193020)
Supplement: Supplementary file 1 [file animals-16-03020-s001.zip › animals-4521016-supplementary.pdf]

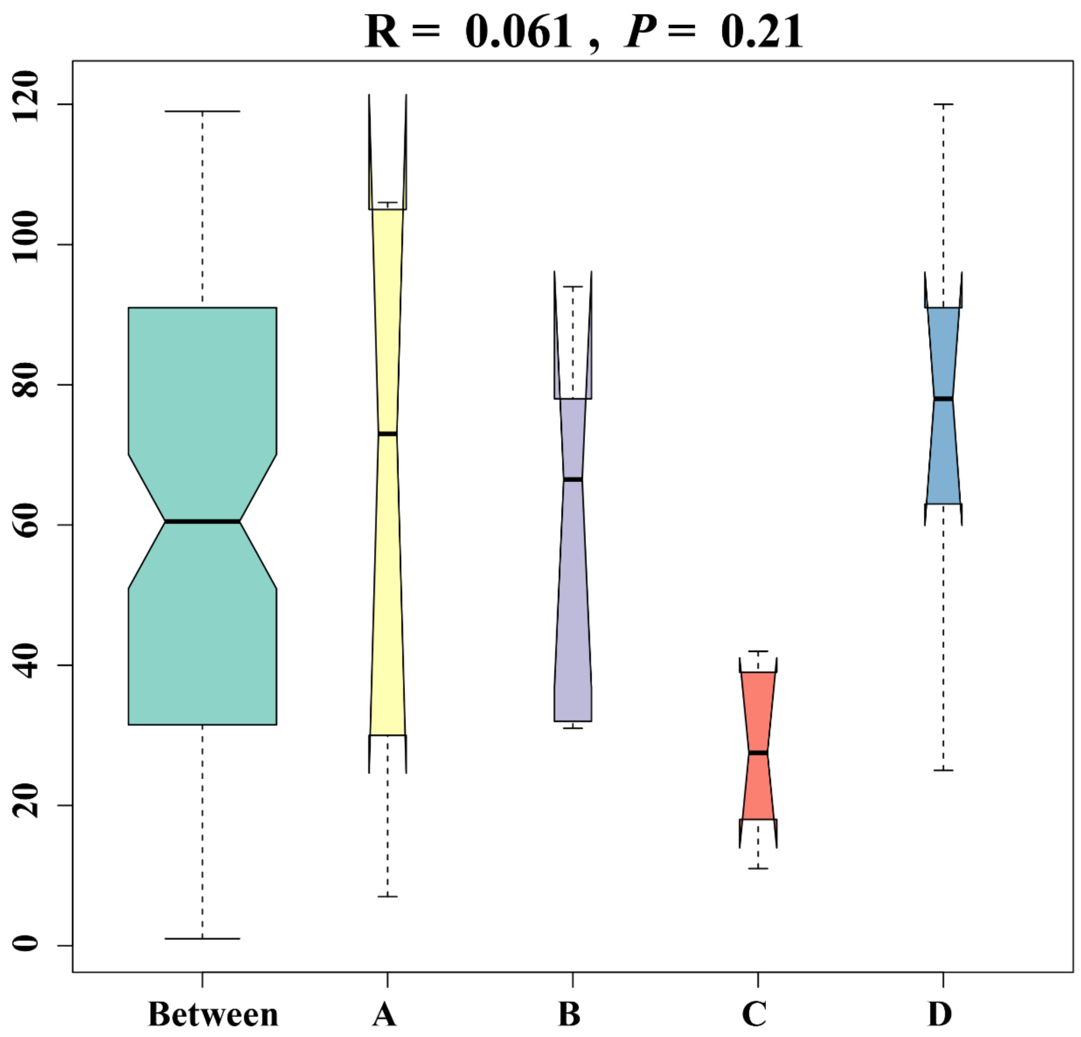

Figure S1. ANOSIM inter-group difference analysis. Box-and-whisker plot illustrating intergroup differences based on ANOSIM. R = 0.061 and *P* = 0.21 represent the ANOSIM R-statistic and significance value, respectively. Boxes indicate the interquartile range, the black horizontal line within each box is the median, and whiskers show the minimum and maximum values of the dataset.
